# Evidence That a TRPA1-Mediated Murine Model of Temporomandibular Joint Pain Involves NLRP3 Inflammasome Activation

**DOI:** 10.3390/ph14111073

**Published:** 2021-10-23

**Authors:** Xenia Kodji, Zizheng Kee, Robyn McKenna, Joao de Sousa Valente, Harriet Ravenscroft, Hayley McMillan, John Gamble, Yvonne Dombrowski, Paul Moynagh, David Brough, Fionnuala T. Lundy, Susan D. Brain, Ikhlas A. El Karim

**Affiliations:** 1 Wellcome-Wolfson Institute for Experimental Medicine, School of Medicine, Dentistry and Biomedical Sciences, Queen’s University Belfast, Belfast BT9 7BL, UK; xenia_kodji@asrl.a-star.edu.sg (X.K.); r.mckenna@qub.ac.uk (R.M.); h.ravenscroft@qub.ac.uk (H.R.); h.mcmillan@qub.ac.uk (H.M.); jgamble14@qub.ac.uk (J.G.); y.dombrowski@qub.ac.uk (Y.D.); paul.moynagh@mu.ie (P.M.); f.lundy@qub.ac.uk (F.T.L.); 2 Section of Vascular Biology and Inflammation Section, School of Cardiovascular Medicine and Sciences, King’s College London, London SE1 9NH, UK; e0441602@u.nus.edu (Z.K.); joao.de_sousa_valente@kcl.ac.uk (J.d.S.V.); 3 Institute of Immunology, Department of Biology, National University of Ireland Maynooth, Eircode W23 F2H6, Ireland; 4 Division of Neuroscience & Experimental Psychology, School of Biological Sciences, University of Manchester, Manchester M13 9PL, UK; david.brough@manchester.ac.uk; 5 Manchester Institute for Collaborative Research on Ageing, School of Social Sciences, Manchester M13 9PL, UK; 6 Lydia Becker Institute of Immunology and Inflammation, University of Manchester, Manchester M13 9PT, UK

**Keywords:** temporomandibular arthritis, TRPA1, NLRP, inflammasome, mouse

## Abstract

This study investigates the role of transient receptor potential ankyrin 1 (TRPA1) in murine temporomandibular joint (TMJ) inflammatory hyperalgesia and the influence of the NLR family pyrin domain-containing 3 (NLRP3) inflammasome. Two distinct murine models of TMJ pain and inflammation (zymosan and CFA) were established. Spontaneous pain-like behaviours were observed as unilateral front paw cheek wipes. Ipsilateral cheek blood flow was used as a measure of ongoing inflammation, which, to our knowledge, is a novel approach to assessing real-time inflammation in the TMJ. Joint tissue and trigeminal ganglia were collected for ex vivo investigation. Both zymosan and CFA induced a time-dependent increase in hyperalgesia and inflammation biomarkers. Zymosan induced a significant effect after 4 h, correlating with a significantly increased IL-1β protein expression. CFA (50 µg) induced a more sustained response. The TRPA1 receptor antagonist A967079 significantly inhibited hyper-nociception. The NLRP3 inhibitor MCC950 similarly inhibited hyper-nociception, also attenuating inflammatory markers. In the trigeminal ganglia, CFA-induced CGRP expression showed trends of inhibition by A967079, whilst lba1 immunofluorescence was significantly inhibited by A967079 and MCC950, where the effect of TRPA1 inhibition lasted up to 14 days. Our results show that stimulation of TRPA1 is key to the TMJ pain. However, the inflammasome inhibitor exhibited similar properties in attenuating these pain-like behaviours, in addition to some inflammatory markers. This indicates that in addition to the therapeutic targeting of TRPA1, NLRP3 inhibition may provide a novel therapeutic strategy for TMJ inflammation and pain.

## 1. Introduction

The temporomandibular joints (TMJ) encompass the joint area that connects the mandibular condyle (jaw) to the temporal bone (skull), consisting of an articular disc and the glenoid fossa. Temporomandibular disorders (TMD) are a heterogenous group of conditions affecting the TMJ and surrounding areas, characterized by chronic joint and muscle pain, limited jaw movement, and clicking [1]. About 30% of TMD sufferers report the painful form of the disorders, of which 65% reported recurrent pain, mainly affecting both the joint and muscle areas [2,3]. The pathophysiology of TMDs is complex and not well understood, although it is thought that one of the main mechanisms of TMD is inflammation, associated with TMD arthritis, that occurs in a similar manner to the development of rheumatoid arthritis (RA) [2]. Despite the recent success in the discovery of novel anti-arthritic treatments, there are no specific drugs available for the treatment of TMD, with some patients failing to respond to available anti-inflammatory therapies, indicating an unmet need for new treatments. The non-selective cation channel transient receptor potential ankyrin 1 (TRPA1) is localized to approximately 65–70% of C and Aδ-fiber nociceptive sensory neurons that also express the TRP vanilloid 1 (TRPV1) [4]. These neurons release CGRP and are involved in the pain pathway [5,6]. We have previously shown a direct link between TRPA1 and the pain associated with joint inflammation [7,8] in our murine models of knee joint arthritis, and also an involvement of IL-1β in the hyper-nociception pathways [9]. By comparison, murine models of TMJ are limited and the sensory mechanisms involved in TMD are poorly characterized. The models have historically been acute rather than mimicking the prolonged periods of pain that are observed in humans. We have established two murine models of TMD to investigate the influence of TRPA1 and potential links with inflammatory hyperalgesia.

In addition to painful symptoms, TMJ arthritis is also strongly associated with increased inflammatory markers, such as IL-1β in the saliva [10] and the joints of TMD patients [11], which has been correlated with pain severity [11], although this is controversial [12]. Additionally, the IL-1Ra gene variant was recently associated with increased risk of developing TMD; hence, mechanisms driving IL-1β production and release are potential targets for TMD treatment [13]. The production of mature IL-1β requires the activation of two pathways: (i) pattern recognition receptors such as Toll-like receptors (TLRs) increase pro-IL-1β expression through NF-κB activation, and (ii) subsequent inflammasome activation which converts pro-IL-1β (~35 kDa) to mature IL-1β (17 kDa), resulting in its release from the cells and pro-inflammatory effects. NLRP3 Inflammasome is an intracellular multiprotein complex, which is activated by danger signals and has recently been shown to be a promising target in inflammation models of pain and gouty arthritis [14,15]. However, precisely how the NLRP3 inflammasome is activated in various disease conditions remains poorly understood. Signals that initiate inflammasome formation can be delivered by environmental irritants or self-derived molecules associated with cell damage [16]. Hence, it is possible that communication via ion channels, such as the polymodal TRPA1, which is activated by a wide range of external and endogenous products generated by tissue damage [17,18], contributes to inflammasome activation. At present it is not known if the NLRP3 inflammasome may contribute to TMJ arthritis or the role of TRPA1 activation, if any, in inflammatory TMJ arthritis. In this study, we have used two inflammatory models to mimic the acute and longer-term TMJ pain and inflammation. The first is zymosan (yeast cell walls) with an inflammatory pathology that is significant after 4 h [19,20]. Zymosan has recently been justified for use in a rat TMD model [20]. We measured spontaneous nociceptive sensation, observed here by front paw wiping of the face [21], and to our knowledge, ipsilateral cheek blood flow is a novel indicator of real-time inflammation. As our group has previously characterized CFA inflammation to induce TRPA1-dependent joint inflammatory arthritis in mice (>14 days) [7], we also established a CFA-induced chronic TMD model. Whilst both models exhibited TRPA1-dependent pain, we also revealed a role for the NLRP3 inflammasome through the use of the selective NLRP3 inflammasome inhibitor MCC950.

## 2. Results

### 2.1. Zymosan Mediates Acute Pain and Inflammation in Murine TMJs

**Figure 1:**
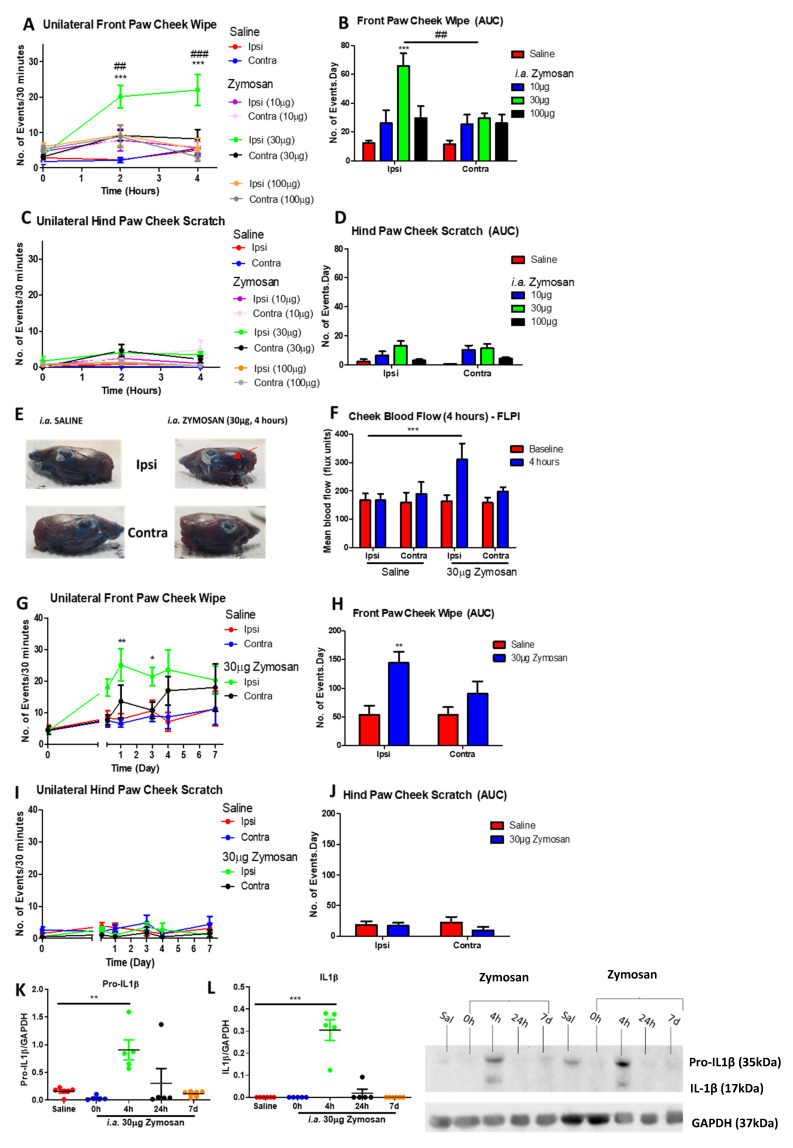
Intraarticular (i.a.) zymosan into the TMJ mediated increase in pain-like spontaneous behaviours and blood flow in vivo. (**A**) Number of unilateral front paw cheek wipe, (**C**) number of unilateral hind paw cheek scratch over 30 min at 2 h or 4 h post-zymosan injection; (**B**,**D**) Area under the curves (AUC) of the corresponding behaviour graphs; (**E**) representative images of oedema formation 4 h post-zymosan injection visualized using the Evans blue dye (red arrow); (**F**) Cheek blood flow as measured by the Full Field Perfusion Imager (FLPI) 4 h following zymosan injection; (**G**) Numbers of unilateral front paw cheek wipe and (**I**) unilateral hind paw cheek scratch over 30 min at various timepoints following i.a. zymosan injection; (**H**,**J**) AUCs of the corresponding graphs, (**K**) Pro-IL1β and (**L**) mature IL-1β Western blots in the TMJ samples. Data represent mean ± SEM, *n* = 6−7 animals per group. Data analysed by one-way ANOVA (**K**,**L**) or two-way ANOVA (repeated measures for **A**,**C**,**G**,**I**) with Bonferroni’s post hoc test.* *p* < 0.05, ** *p* < 0.01, *** *p* < 0.001 for saline vs. zymosan, ## *p* < 0.01, ### *p* < 0.001 between zymosan ipsi vs. contra.

The model was initially characterized by ensuring that intra-articular (i.a.) injection of zymosan into the TMJ caused reproducible responses in the mouse. Zymosan (30 µg i.a.) produced an increase in unilateral front paw cheek wipes (associated with painful sensations) 2–4 h post-injection (Figure 1A,B) in the ipsilateral cheek when compared to the saline-treated group. This compared with a slight, but not significant, increase in front paw wiping observed in the contralateral side (Figure 1A,B). By comparison, no significant changes in hind paw cheek scratching were observed in response to this dose of zymosan (Figure 1C,D). These results highlighted that this treatment induced a painful, but not itching, phenotype. Of note, other doses of zymosan (10 µg or 100 µg) failed to induce any significant pain-like behaviours and were not further investigated. Hence, zymosan (30 µg) was herein used to induced TMJ inflammation and pain in further experiments.

To determine if the zymosan injection was indeed mediating inflammation in the TMJ and surrounding muscular areas, Evans blue dye, which binds non-covalently to plasma albumin, was used to determine areas where inflammatory oedema formation had occurred (Figure 1E, red arrow). Indeed, 4 h following zymosan (i.a. 30 µg), oedema (blue areas) was observed in the muscular areas around the TMJ, in the ipsilateral, but not contralateral side; however, it was not possible to quantify this localised oedema formation. Saline (i.a.) did not induce oedema on either side, confirming the specific inflammatory response to zymosan injection and not to needlestick injury. To further characterize inflammation in the area, cheek blood flow was measured using the laser speckle technique (FLPI). Indeed, increased cheek blood flow was observed at 4 h following zymosan (30 µg) injection in the ipsilateral cheek, further confirming the development of inflammation with this treatment regime (Figure 1F). The duration of zymosan-induced pain was ipsilateral-specific during the first 3 days following injection, before the results became variable and the contralateral side began to show cheek wiping (Figure 1G,H). In contrast, there were no significant differences in cheek scratching (Figure 1I,J), confirming the specificity of this response to pain-like sensations over the entire 7-day period.

To confirm inflammation, Western blots were performed in the collected TMJ samples for pro-IL-1β and mature IL-1β. Pro-IL-1β and mature IL-1β expression was significantly increased 4 h post-30 µg zymosan injection and the signal was reduced from 24 h onwards (Figure 1K,L). Hence, zymosan (30 µg i.a.) and the 4 h timepoint were chosen as the acute TMJ model to investigate the inflammation and pain for future experiments.

### 2.2. TRPA1 and NLRP3 Antagonists Have Differential Effects on Zymosan-Mediated Spontaneous Behaviours and Inflammation

**Figure 2:**
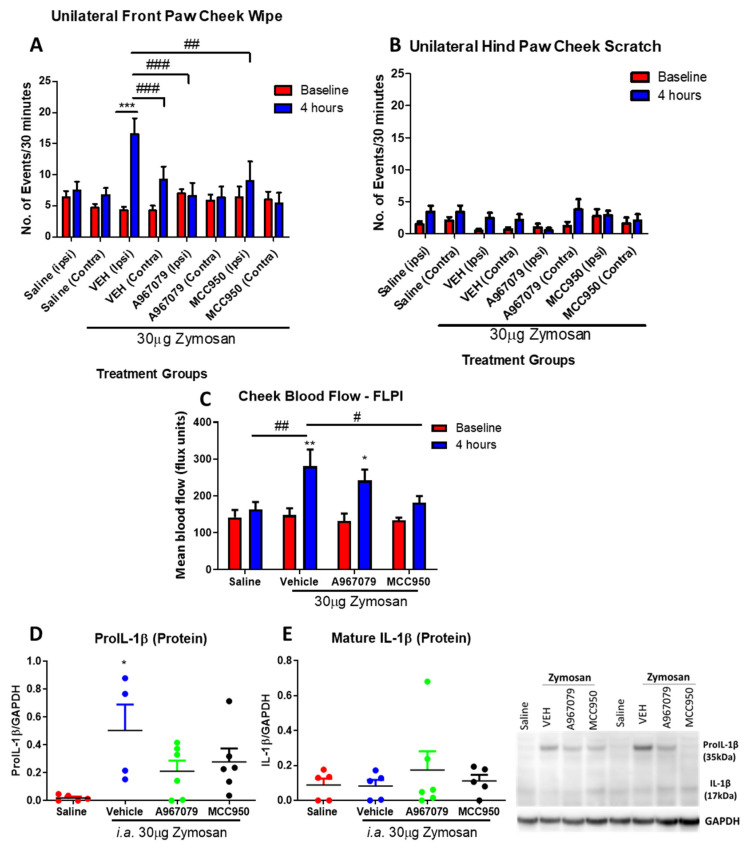
The distinct effects of a TRPA1 antagonist (A967079) or an NLRP3 antagonist (MCC950) on the observed spontaneous behaviors and blood flow in the acute 4 h zymosan model. Number of (**A**) unilateral front paw cheek wipe and (**B**) unilateral hind paw cheek scratch in response to saline or zymosan when pre-treated with vehicle (10% DMSO, 10% Tween-80 in saline), A967079 (100mg/kg, i.p.) or MCC950 (10mg/kg, i.p.), (**C**) cheek blood flow as measured by the FLPI, (**D**) Pro-IL1β and (**E**) mature IL-1β Western blots of the TMJ samples. Data represent mean ± SEM, *n* = 7−14 animals per group. Data analysed by two-way ANOVA with Bonferroni’s post hoc test. * *p* < 0.05, ** *p* < 0.01, *** *p* < 0.001 between saline vs. zymosan as indicated, # *p* < 0.05, ## *p* < 0.01, ### *p* < 0.001 between the indicated groups.

Pre-treatments with either a TRPA1 antagonist (A967079) or NLRP3 antagonist (MCC950) resulted in a significant reduction in zymosan-induced front paw cheek wiping as we predicted, indicative of analgesic activity. In contrast, no effects were observed in hind paw scratching, suggesting that the effects were specific for the pain sensation, and not due to the treatments affecting general motor functions or activities. Both antagonists reduced the pain-associated behaviours to a similar extent (Figure 2A,B). By comparison, although there was a trend of decreases in the blood flow response in both MCC950- and A967079-treated mice, only that in MCC950-treated mice reached significance after 4 h, indicative of an effect in inhibiting the inflammatory component (Figure 2C). There was a significant increase in Pro-IL-1β protein expression in the vehicle-treated zymosan group, with minimal expression observed in mature IL-1β (Figure 2D,E). There were trends, but not a significant reduction in Pro-IL-1β expression with both TRPA1 and NLRP3 antagonist treatments (Figure 2D).

### 2.3. Complete Freund’s Adjuvant (CFA) Injection into the TMJ Induced Sustained Spontaneous Pain and Increased Blood Flow in Mice

**Figure 3:**
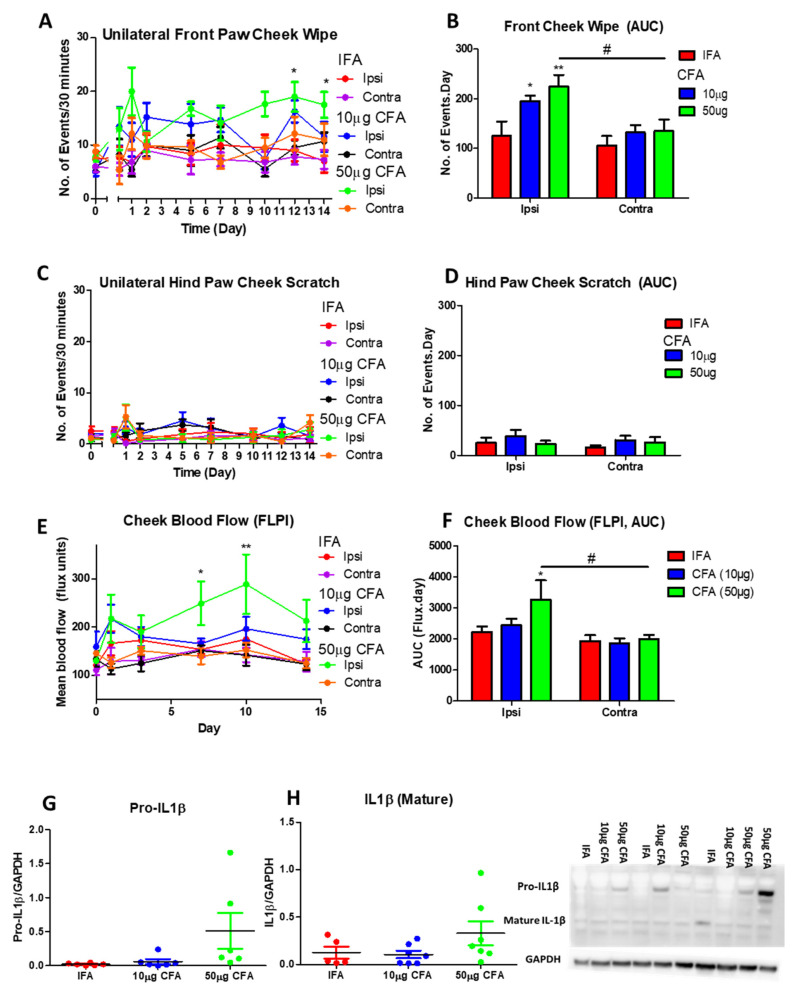
Fifty μg CFA injection i.a. into the TMJ resulted in a significant, consistent increase in pain-like spontaneous behaviours and cheek blood flow over 14 days. The numbers of (**A**) unilateral front paw cheek wipe and (**C**) unilateral hind paw cheek scratch observed over 30 min. AUC for the (**B**) front paw cheek wipe and (**D**) hind paw cheek scratch calculated over the 14 days. (**E**) Cheek blood flow measured using the FLPI and (**F**) AUC of the cheek blood flow calculated over 14 days, (**G**) ProIL-1β and (**H**) mature IL-1β Western blots on TMJ samples. Data represent mean ± SEM, *n* = 6−7 animals per group. Data analysed by two-way ANOVA (repeated measures for figures (**A**,**C**,**E**) with Bonferroni’s post hoc test. * *p* < 0.05, ** *p* < 0.01 between IFA and CFA, # *p* < 0.05 between ipsi vs. contra.

To characterize a more clinically relevant model, we investigated a longer-term model of TMJ pain and inflammation by injecting CFA into the TMJ and observing the in vivo parameters over 2 weeks. Two different doses were initially tested to determine the best concentration of CFA to produce a robust pain and inflammatory response. CFA (50 μg) induced a significant increase in spontaneous pain behaviours, but not scratching behaviours (Figure 3A–D), as well as increased cheek blood flow (Figure 3E–F), whereas minimal changes were observed in response to CFA (10 μg) injection (Figure 3A–F). The protein expression was studied over 14 days and there was a trend towards increased Pro-IL-1β and mature IL-1β expression with CFA (50 µg) (Figure 3G,H). Due to the minimal effects observed on the contralateral side, our future studies focused on the hyperalgesia and inflammation on the ipsilateral side.

### 2.4. TRPA1 and NLRP3 Antagonists Inhibit the Spontaneous Pain Mediated by CFA, However, Have Differential Effects on Cheek Blood Flow and Inflammatory Markers

**Figure 4:**
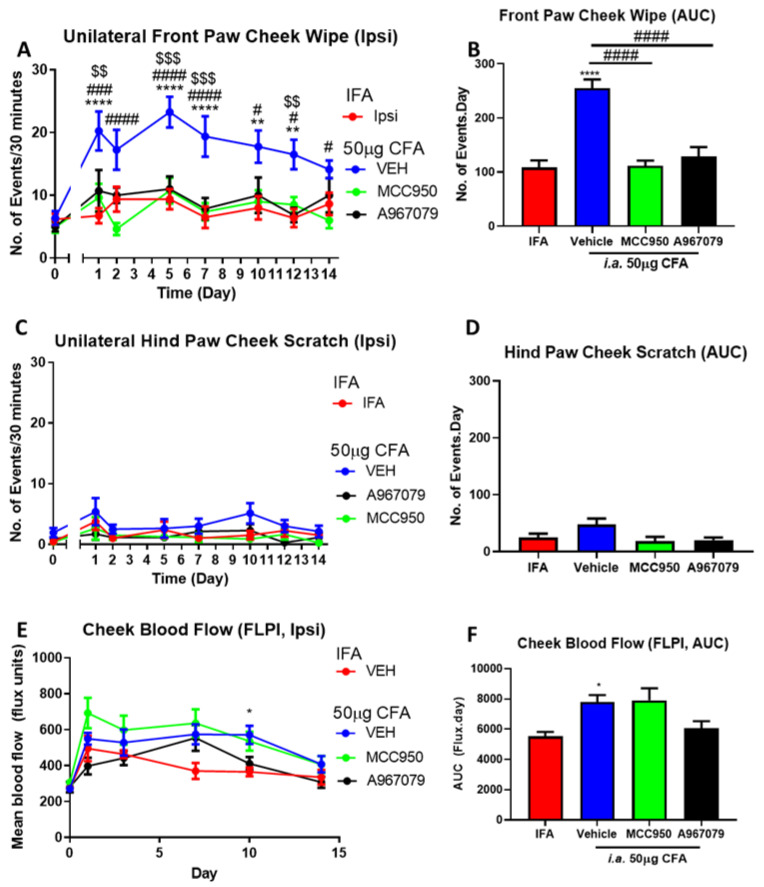
Repeated TRPA1 antagonist (A967079) and NLRP3 antagonist (MCC950) over 14 days significantly inhibited CFA-mediated pain-like behaviours and have differential effects on cheek blood flow. The numbers of ipsilateral (**A**) unilateral front paw cheek wipe and (**C**) hind paw cheek scratch observed over 30 min. The changes in cheek blood flow was observed at various timepoints (**E**). The AUC for (**B**) front paw cheek wipe, (**D**) hind paw cheek scratch, and (**F**) cheek blood flow. Data represent mean ± SEM, *n* = 7−8 animals per group. Data analysed by two-way repeated measures ANOVA (**A**,**C**,**E**) or one-way ANOVA (**B**,**D**,**F**) with Bonferroni’s post hoc test. * *p* < 0.05, ** *p* < 0.01, **** *p* < 0.0001 IFA vs. CFA, # *p* < 0.05, ### *p* < 0.001, #### *p* < 0.0001 CFA/VEH vs. CFA/MCC950 in the timepoint reading or between indicated groups, $$ *p* < 0.01, $$$ *p* < 0.001 between CFA/VEH vs. CFA/A967079 in the timepoint reading.

The effects of the TRPA1 and NLRP3 antagonists, which previously showed significant reduction in pain-like behaviours and inflammatory markers in response to the acute zymosan treatment in the TMJ were then investigated in a more sustained model of CFA-induced TMJ. The administration of the antagonists was adapted to daily treatments to achieve efficacy. The repeated treatments with either a TRPA1 antagonist (A967079 i.p.) or an NLRP3 antagonist (MCC950 i.p.) resulted in significant reduction in the front paw cheek wipe on the ipsilateral side (Figure 4A,B) in each case when compared with vehicle. No significant effects on the scratching behaviours were evident (Figure 4C,D), consistent with the observations during the CFA model characterization (Figure 3). While there is a consistent increase in the cheek blood flow in response to CFA injection over the 14-day observation, neither of the treatments had any significant effect on blood flow, although a trend of reduction was observed in the A967079-, but not the MCC950-, treated group (Figure 4E,F).

**Figure 5:**
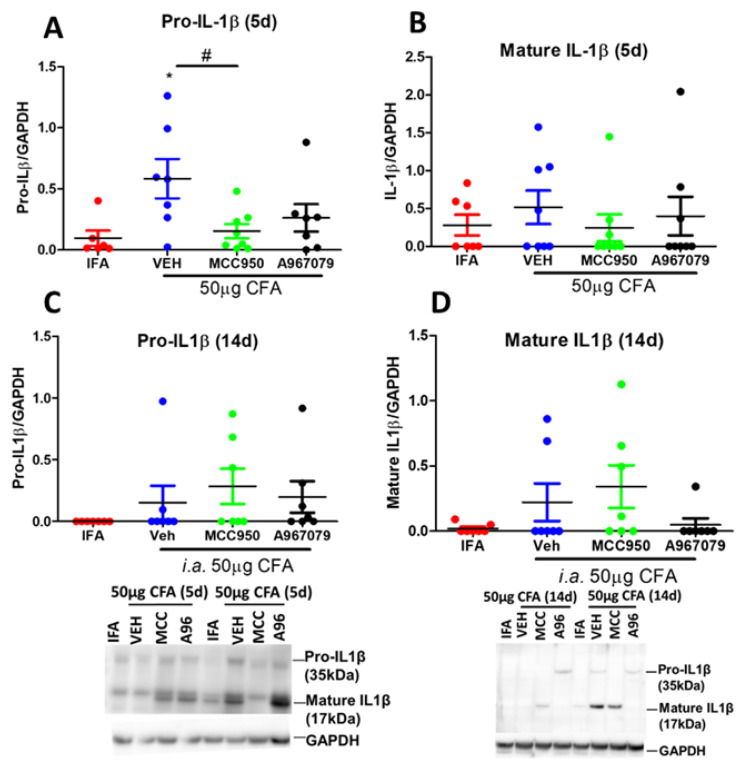
Western blotting for inflammasome-related markers in CFA-injected TMJs collected at days 5 or 14 in response to repeated treatments with a TRPA1 antagonist (A967079) or an NLRP3 antagonist (MCC950). (**A**,**C**) ProIL-1β in TMJs collected at days 5 or 14; (**B**,**D**) Mature IL-1β in TMJs collected at days 5 or 14. Below are the representative blot images. Data represent mean ± SEM, *n* = 7−8 animals per group. Data analysed by one-way repeated measures ANOVA with Bonferroni’s post hoc test. * *p* < 0.05 IFA vs. CFA, # *p* < 0.05CFA/VEH vs. CFA/MCC950.

To further characterize the inflammatory response in the CFA model, we measured IL-1β expression and release at days 5 and 14. Pro-IL-1β protein expression showed a more significant increase at day 5 post-CFA treatment, a significant reduction with MCC950 and an inhibitory trend with A967079 (Figure 5A). Although the levels of mature IL-1β were raised with CFA, no significant reductions were observed with MCC950 treatment (Figure 5B). At day 14, a lower and more variable expression of Pro-IL-1β and mature IL-1β was observed, but there was no significant inhibitory effect of MCC950 or A967079 (Figure 5C,D). We noticed no change in NLRP3 expression at this time point too (Supplementary Figure S1).

### 2.5. TRPA1 and NLRP3 Antagonists Modify CFA-Induced Iba1+ Expression in the Trigeminal Ganglia of Treated Mice

**Figure 6:**
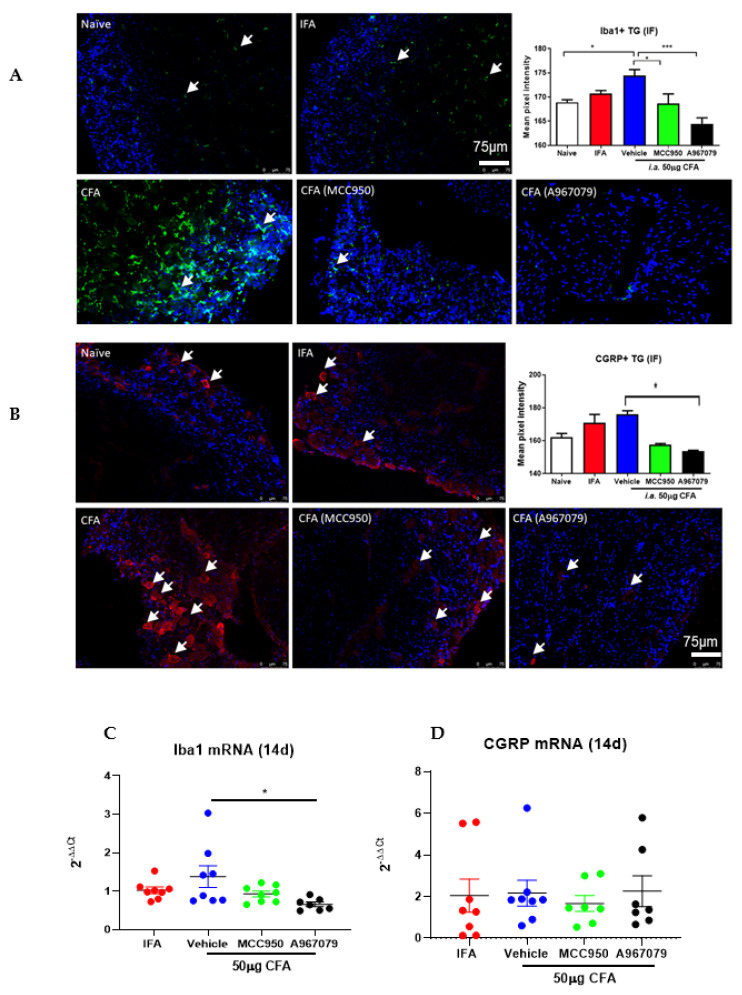
The effect on distinct alteration of CGRP and Iba1 protein and mRNA expression in TG collected at different timepoints post-CFA treatment. (**A**) Representative images and quantification of Iba1+ staining in naïve, IFA and CFA-treated animals collected at day 5 in absence or presence of the NLRP3 (MCC950) and TRPA1 (A967079) antagonists, showing significant reduction in Iba1 with both inhibitors (**B**) Representative images and quantification of CGRP+ staining in naïve, IFA and CFA-treated animals collected at day 5 in presence or absence of NLRP3 (MCC950) and TRPA1 (A967079) antagonists, showing that only A967079 significantly reduced CGRP expression in CFA treated mice. (**C**) Iba1 mRNA expression and (**D**) CGRP mRNA expression in TGs of IFA- or CFA-treated animals in absence of the NLRP3 (MCC950) and TRPA1 (A967079) antagonists. The TGs were collected at 14 days post-CFA treatment. *n* = 7−8 animals. The white arrows highlight the Iba1+ (green) or CGRP+ (red) staining. Scale bar = 75 μm. Data represent mean ± SEM, ordinary one-way ANOVA with Tukey’s multiple comparisons test. Per group, * *p* < 0.05, *** *p* < 0.001.

It has been established that the orofacial pain linked to TMJ is associated with the upregulation in activity of trigeminal nociceptors. To further understand mechanisms involving the trigemino-sensory system, we characterized the protein and mRNA expression of Iba1, the macrophage marker, and CGRP, indicative of neuronal sensitization, in the trigeminal ganglia. Our results showed that Iba1 protein expression increased significantly in the TGs of CFA-treated animals at day 5, while the mRNA expression of this target showed a slight trend of increase at day 14 (Figure 6A,C). Indeed, both MCC950 and A967079 treatments significantly inhibited Iba1 expression at day 5 (Figure 6A), although only A967079 significantly inhibited its expression on day 14 (Figure 6C). On the other hand, the CGRP expression appeared to be slightly increased in the CFA-treated group at day 5, with minimal changes observed in the mRNA at day 14 (Figure 6B,D). Both MCC950 and A967079 showed some trends to reduce CGRP expression at day 5, albeit not significantly, while minimal effects were observed at day 14 (Figure 6B,D). These results suggest that Iba1+ macrophages may play an important role in contributing to the CFA-induced hyperalgesia, and it can be partially targeted by both NLRP3 and TRPA1 antagonists.

## 3. Discussion

In the present study, we used two distinct unilateral models of TMJ inflammatory hyperalgesia, relevant to arthritis and obtained similar results to show: (i) ipsilateral pain-related behaviours (hyper-nociception) as measured by monitoring front paw wiping, (ii) increased markers of TMJ inflammation as measured by a non-invasive technique to measure cheek skin blood flow as well as pro-IL1β/mature IL-1β expression, and (iii) for the first time, we show a dependence of the hyper-nociception and inflammatory phases on TRPA1 and NLRP3 in mouse TMD models. In these studies, it was important to give the TRPA1 antagonist and the inflammasome inhibitor that were investigated systemically, as our research is aimed at searching for new therapeutic agents for this disease, where there is an unmet clinical need.

Among other TRP channels, TRPA1 plays an important role in inflammatory pain [22,23]. To investigate the involvement of TRPA1 in these models, we utilized the selective TRPA1 antagonist (A967079). We have previously shown that the TRPA1 antagonist acts over 5 days to modulate a cutaneous inflammation in a murine model associated with pain-related behaviours [24,25]. In our current study, pre-treatment with the TRPA1 antagonist inhibited the hyper-nociception associated with paw wiping in both the zymosan and CFA models studied. This emphasizes the importance of neuronal TRPA1 in mediating TMD-induced pain. However, there seems to be a distinct difference between the effects of TRPA1 antagonist in the acute zymosan model versus in the longer-term CFA model. In the acute model, the TRPA1 antagonist was less effective in altering the inflammatory markers, be it in the functional (blood flow) measurement or in terms of the IL-1β pathway. This is in keeping with the knowledge from both our group and others that TRPA1 is an environmental sensor, stimulated by a range of noxious stimuli to activate CGRP-containing sensory neurons leading to mechano-transduction and pain signalling [22,26]. Importantly, the pharmacological blockade of TRPA1 attenuates the inflammatory hyperalgesia observed in murine models of knee joint arthritis [7,8]. Indeed, it has been suggested that TRPA1 antagonists are promising as novel therapeutic agents that target pain relief [22]. There are no studies, to our knowledge, where a murine model of TMJ inflammatory hyperalgesia has been investigated for a selective role for TRPA1. We used two models here in order to confirm the relevance of TRPA1 in TMD-induced pain/arthritis. Independently, a combined TRPA1 antagonist and the selective inhibitor of the N-type voltage-gated calcium channels has very recently been shown to attenuate TRPA1-mediated facial grooming in a CFA-induced rat TMJ model [27], and our results build on that finding. A link between zymosan and TRPA1-induced nociception is also established in inflammatory models associated with pain [28,29]. Furthermore, it has been reported that pain is linked to zymosan-induced TLR2/6 activation of inflammation pathways [30,31]. By comparison, CFA-induced inflammatory hyperalgesia has been established to be linked to TRPA1 pain activated via stimulating cell-mediated immunity [32].

We then questioned whether the inflammasome may be involved in TMJ-induced inflammatory hyperalgesia associated with arthritis. It was established that the NLRP3 inflammasome plays a central role in the uncompromising pain associated with gouty arthritis [33] in 2016, and also in arthritic-type components of Chikungunya disease [34], while recently it has been shown to play a role in migraines [35]. In the current study, the NLRP3 inflammasome inhibitor MCC950, which acts selectively to block the conformation of active NLRP3 proteins [36], inhibited both the acute and chronic hyper-nociception, and of importance, showed similar efficacy to the TRPA1 antagonist in these in vivo models. Additionally, MCC950 also showed some inhibitory effects on inflammatory symptoms. On realizing these results, we considered that mechanistic links between the TRPA1 and inflammasome pathways may exist. To pursue this line of enquiry, we investigated whether IL-1 levels were raised in these models. We showed that NLRP3 inhibition significantly reduced pro-IL-1β generation in the TMJs of the zymosan model at 4 h and mature IL-1β production at 5 days in the CFA model, suggesting a reduction in ILB in acute rather than chronic CFA models. Although the effect of TRPA1 antagonist showed similar trends of reduction in both models, it is less significant. Hence, our findings suggest that both TRPA1 and NLRP3 may be overlapping in their mechanisms driving TMJ inflammation and pain via an IL-1β-mediated pathway.

We next investigated the trigeminal ganglia, which innervates the oro-facial sensory nerves, and which are known to be activated as well as sensitized by CFA in rats [37]. We collected the ganglia after 5 days and 14 days from the CFA model to learn more about the potential influence of treatment with the TRPA1 antagonist and inflammasome inhibitor. The CFA model showed a trend of increased levels of the major sensory neuropeptide CGRP and Iba1 expression, both of which are indicative of neuronal sensitization and inflammation, which have been associated with pain. Interestingly, the TRPA1 antagonist was able to inhibit both Iba1 expression (a marker of microglia and inflammation, macrophages) and CGRP, to a less significant extent, on days 5 and 14. This is consistent with the finding that CGRP expression showed trends of reduction by the TRPA1 antagonist and that TRPA1 activation mediates nociception via CGRP release [22,26], although to our knowledge, this is the first study to link the application of TRPA1 antagonist and the modulation of Iba1 expression in the trigeminal ganglia. In contrast, the NLRP3 antagonist only had a significant effect on Iba1 expression over the 5 days post CFA treatment. MCC950 was previously shown to be able to reduce CGRP and IL-1β expression in the trigeminal ganglia of a murine migraine model [35] and reduce Iba1 expression in the hippocampus of aged mice with neurocognitive disorders [38]. Our study is the first to indicate that MCC950 is able to modulate the expression of Iba1 expression in the trigeminal ganglia and a possible mechanism in contributing to TMD-associated pain.

## 4. Materials and Methods

### 4.1. In Vivo Induction of Temporomandibular Joint (TMJ) Inflammation and Arthritis

All animal procedures were carried out in accordance with the UK Home Office Animals (Scientific Procedures) Act 1986 and the ARRIVE guidelines. A total of 140 mice were used in this study. This study was approved by the King’s College Animal Care and Ethics Committee. Male CD1 mice (6–8 weeks, Charles River, Harlow, UK) were used in this study. All mice were maintained in a climatically controlled environment (22 °C) and exposed to a 12/12h light/dark cycle. All recovery procedures were performed under 2% isoflurane (Isocare; Animalcare, York, UK) with 2% oxygen. All procedures were terminated by cervical dislocation. The animals were randomized at the start of the procedure, while the investigators were blinded to the treatment groups. The areas around the TMJ were shaved and acute TMJ inflammation and pain was induced ipsilaterally by intraarticular (i.a.) injection of 10 µL of Zymosan A (10, 30, or 100 µg from *S. cerevisiae*; Sigma-Aldrich, Gillingham, UK) or vehicle (saline) using a BD Microfine^+^ 30 G × 8 mm needle attached to an insulin syringe (Beckton, Dickinson, West Sussex, UK) into the TMJ capsule, as previously described [39]. The zymosan model was investigated and tested for a maximum of 7 days. For the chronic model of TMJ inflammation, 10 µL of Complete Freund’s Adjuvant (CFA, 5 mg/mL, Chondrex, US or 1 mg/mL stock, Sigma-Aldrich, Gillingham, UK) or vehicle control (Incomplete Freund’s Adjuvant/IFA) was injected ipsilaterally using the same technique. Subsequent measurements were carried out for up to 14 days before terminating the study.

#### 4.1.1. Blood Flow Measurement of the TMJ and Surrounding Areas

To provide an estimation of the inflammation, blood flow was measured in an area around the mouse cheek area under recovery anaesthesia using the Full Field Laser Perfusion Imager (FLPI; Moor Instruments, Axminster, UK) in real time. Mouse body temperature was maintained at 36 °C using a homeothermic mat throughout the measurement. The scanner was placed 20 cm above the cheek. The scanner emitted a laser that penetrated to a 1–2 mm depth of the measured area and was successfully used to measure the blood flow in mouse knee joints [8]. The settings used were as follows: high resolution capture (25 frames, 1 s/frame), gain: auto, exposure: 8.3 ms. The imaging was initiated after ~2 min or later following the shaving of the cheek area. The blood flow was measured until a stable blood flow reading was obtained for 5 min continuously and the mean blood flux units (proportional to the blood flow) were calculated using the MoorFLPI Review 3.0 software (Moor Instruments) for the last 2 min of measurement. Regions of interest were highlighted with a line around the treated area and kept consistent throughout the measurement period.

#### 4.1.2. Spontaneous Behaviour Observations

Mice were acclimatized to the procedure room for at least 1 h before observations and baseline measurements were taken over 2 days to habituate them before disease induction. The baseline readings over 2 days were averaged as “day 0” values. The observations were carried out in a see-through behaviour chamber (20 × 20 × 14 cm^3^, Ugo Basile, Gemonio VA, Italy) where the animals were allowed to further habituate to the observation chamber for 15 min before recording the observation. Spontaneous behaviours were recorded for 30 min at each timepoint, observing for behaviours indicative of pain (unilateral front paw cheek wiping) and itch (unilateral hind paw cheek scratching) as previously described [21].

#### 4.1.3. Pharmacological Treatments

For the acute zymosan studies, the same vehicle (10% DMSO, 10% Tween-80 in saline) was used for both A967079 (Alomone Labs, Israel) ((1E,3E)-1-(4-Fluorophenyl)-2-methyl-1-pentene-3-one oxime), a selective TRPA1 antagonist [40], or MCC950 (Insight Biosciences, UK) (N-[[(1,2,3,5,6,7-hexahydro-s-indacen-4-yl)amino]carbonyl]-4-(1-hydroxy-1-methylethyl)-2-furansulfonamide), a selective NLRP3 antagonist [41]. The drugs were administered (i.p., 100 mg/kg) 30 min prior to the injection of zymosan. For longer-term CFA studies, A967079 or MCC950 was administered i.p. at 100 mg/kg or 10 mg/kg, respectively, 30 min prior to CFA injection at day 0. A967079 was then administered once daily at 60 mg/kg i.p. thereafter, while MCC950 was administered i.p. once daily (10 mg/kg) on days 1, 2, then every 2 days thereafter until the end of the study period. These respective treatment regimens were chosen as they previously showed to be effective in various pain and inflammatory murine models [40,41].

#### 4.1.4. Using the Evans Blue Dye for Observing the Development of Oedema in the TMJ

To determine the formation of oedema in the TMJ, the mice were anaesthetized under recovery anaesthesia. Plasma extravasation was quantified using Evans Blue, a dye that binds to plasma albumin, to indicate oedema formation in the affected area. Mice received Evans Blue (25%; 2.5 mg/g of animals, i.v.), prior to being shaved in the cheek area and receiving i.a. injection of either zymosan or saline in the ipsilateral cheeks [42]. The contralateral cheeks received no treatment. The mice were allowed to recover for 4 h before terminating the study and the facial region was dissected to visualize the area where oedema had formed.

### 4.2. Western Blotting

The TMJs were homogenized in SDS lysis buffer (50 mM Tris base pH 6.8, 10% glycerol, 2% SDS) containing a HALT protease/phosphatase inhibitor cocktail (Thermo Fisher Scientific, Loughborough, UK). The TMJ samples were homogenized using a hand-held homogenizer (Ultra Turrax T25, IKA Laboratories, Oxford, UK) at maximum speed for 2 min, cooled after 30 s of homogenization. This cycle was repeated until the tissues were fully pulverized. The samples were then centrifuged, and supernatants were collected and assayed. A total of 50 µg of protein per sample was prepared and ran in an electrophoresis chamber (Thermo Fisher Scientific, Loughborough, UK) and was transferred and immobilised onto Immun-Blot^®^ PVDF membranes (Bio-Rad Laboratories, Hertfordshire, UK) at 30 V for 1 h. Non-specific binding was blocked in 5% BSA before the membrane was washed in PBS containing 0.1% Tween-20 between incubations. Membranes were incubated in primary antibodies overnight at 4 °C and secondary antibodies at RT for 1 h. The membrane was then incubated with 1ml of enhanced chemiluminscence solution (Luminata Crescendo Western HRP substrate, Millipore, Watford, UK) and was developed in the G-Box Gel Documentation System (Syngene International, Bengaluru, India). Chemiluminescence images were captured with Syngene 2D Gel Imaging software and densitometry analyses were performed on the ImageJ software with arbitrary intensity values expressed as a ratio to the values for GAPDH loading control. Rat mAb anti-human/mouse NLRP3/NALP3 (MAB7578, 1:1000, R&D Systems, Abingdon, UK) Goat pAb anti-mouse IL1β/IL-1F2 antibody (AF-401-NA, 1:800, R&D Systems, Abingdon, UK) Anti-mouse glyceraldehyde-3-phosphate dehydrogenase mAb were used for loading control (GAPDH; AM4300; 1:2000, Thermo Fisher Scientific, Loughborough, UK). The secondary antibody (HRP-conjugated) used was: (1) goat anti-mouse IgG antibody (AP132P, 1:2000, Millipore, Watford, UK).

### 4.3. Quantitative rt-PCR

RNA extraction was performed in the trigeminal ganglia from Zymosan and CFA models. Trigeminal ganglia were homogenised using the stainless steel bead-based TissueLyser II system (Qiagen, Manchester, UK) and total RNA was extracted using the RNAEasy kit (Qiagen, Manchester, UK), according to the manufacturer’s instructions. The RNA concentration as well as quality were measured using the Nanodrop 2000 Spectrophotometer where absorbance ratios for the A260/280 and A260/230 values between 1.8–2.2 were deemed acceptable. A total of 500 ng of RNA was reverse transcribed using the Superscript ViLO cDNA synthesis reagent (Invitrogen, Loughborough UK). qPCR was performed in triplicate using the 7900HT Real-Time PCR instrument (Applied Biosystems, Massachusetts, USA). PowerUp SYBR Green Master Mix kit (Thermo Fisher Scientific, Loughborough, UK) was used, and the reaction settings used were as per manufacturer’s instructions. The melt curve was analysed to ensure product specificity after each amplification. Primers design was performed according to the MIQE guidelines [43] and details previously described [44].

### 4.4. Immunofluorescence Staining

Trigeminal ganglia (TGs) were dissected and fixed in 4% paraformaldehyde for 4 h at 4 °C before being stored in 20% sucrose solution overnight at 4 °C. These samples were then embedded into OCT and blocks were stored at −80 °C until further processing. Sections of 20 μm were processed on a cryostat (CM1900, Leica Biosystems, Milton Keynes, UK). Sections were allowed to dry for 1 h at room temperature before rinsing the slides in TBS-0.25% tween 20 (TBST) for 10 min. The sections were then permeabilized in TBST-0.3% Triton-X for 30 min before further rinsing and blocking in 10% goat serum in TBST for 1 h at room temperature. Primary antibodies used were rabbit anti-Iba1 (1:500, 019-19741, Wako Chem, Osaka, Japan) and sheep anti-CGRP (1:100, BML-CA1137-0100, Enzo Life Sciences, Exeter, UK) and were incubated overnight at 4 °C. Following rinsing, sections were incubated at room temperature for 1–2 h in secondary antibodies: AlexaFluor^®^488 Goat anti-rabbit IgG (1:200, A11008, Thermo Fisher Scientific, Loughborough, UK), AlexaFluor^®^594 donkey anti-sheep IgG (1:200, A11016, Thermo Fisher Scientific, Loughborough, UK) before a final rinse and mounting the coverslips using the ProLong™ Gold Antifade mountant with DAPI (P36931, Thermo Fisher Scientific, Loughborough, UK) and slides were allowed to dry overnight at room temperature. The sections were imaged on the Leica DM5500 microscope (Leica Biosystems, Milton Keynes, UK) and the acquisition parameters remained constant for all of the imaged samples.

### 4.5. Quantification of Iba1 and CGRP Immunofluorescence

Iba1 and CGRP immunofluorescence intensity for each treatment group was quantified using the Fiji Image J software. All images were taken at ×20 magnification and exposure settings were kept constant to ensure that reliable comparisons could be made between treatment groups. Images were first de-speckled to reduce noise. Images were then converted to greyscale and upper and lower thresholds were set to exclude background and bright artefacts. Particle analysis was used to measure the mean pixel intensity across all images.

### 4.6. Statistics

Data were represented as mean ± SEM and were analysed on Prism 8 software (GraphPad Software, San Diego, CA, USA). Repeated measures, one-way, or two-way ANOVA was performed with Bonferroni’s or Tukey post hoc test, as appropriate. A value of *p* < 0.05 was considered statistically significant. The area under the curve analysis was calculated on the Prism 5 software (GraphPad Software, San Diego, CA, USA) and the unit was defined as No. of events.day (for the behavioural observations) or flux.day (for the cheek blood flow). For experiments involving drug treatments, we used the same vehicle group, as the drugs were made up in the same vehicle and this allowed us to reduce the number of mice used overall, according to the 3Rs principles.

## 5. Conclusions

In conclusion, both NLRP3 and TRPA1 have a critical but distinct role in the pain observed in this model. The results clearly demonstrate that TRPA1 is key in mediating the hyper-nociception acting mainly within the neuronal component of the orofacial sensory nerves and is in keeping with the concept that TRPA1 plays a primary role in mediating TMD-induced pain. We made the discovery that the inflammasome inhibitor MCC950 has a similar inhibitory effect on hyper-nociception and a more significant role in altering the underlying IL-1β-mediated inflammation in the TMJ, which then activates the appropriate sensory nerve pathways that contribute to pain. Thus, we identify drug targets for two pathways that ameliorate the hyper-nociception during both the development and maintenance phases in these models, each important for further investigation, particularly the formation of the inflammasome, in addition to the activation of the sensory nerves by TRPA1.

## Data Availability

Data is contained within the article and Supplementary Material.

## Supplementary Materials

The following are available online at https://www.mdpi.com/article/10.3390/ph14111073/s1, Figure S1: Western blotting for NLRP3 expression in CFA-injected TMJs collected at days 5 or 14 in response to repeated treatments with a TRPA1 antagonist (A967079) or an NLRP3 antagonist (MCC950). A, B) NLRP3 in TMJs collected at days 5 or 14; Figure S2: Proposed cross talks of TRPA1 and NLRP3 inflammasome in CFA induced TMJ inflammation and pain.
