# Supplementary material for: Evidence That a TRPA1-Mediated Murine Model of Temporomandibular Joint Pain Involves NLRP3 Inflammasome Activation
Source: Pharmaceuticals (Basel). 2021 Oct 23;14(11):1073. doi: 10.3390/ph14111073 (PMC8622821; doi:10.3390/ph14111073)
Supplement: Supplementary file 1 [file pharmaceuticals-14-01073-s001.zip › pharmaceuticals-1362646-supplementary.pdf]

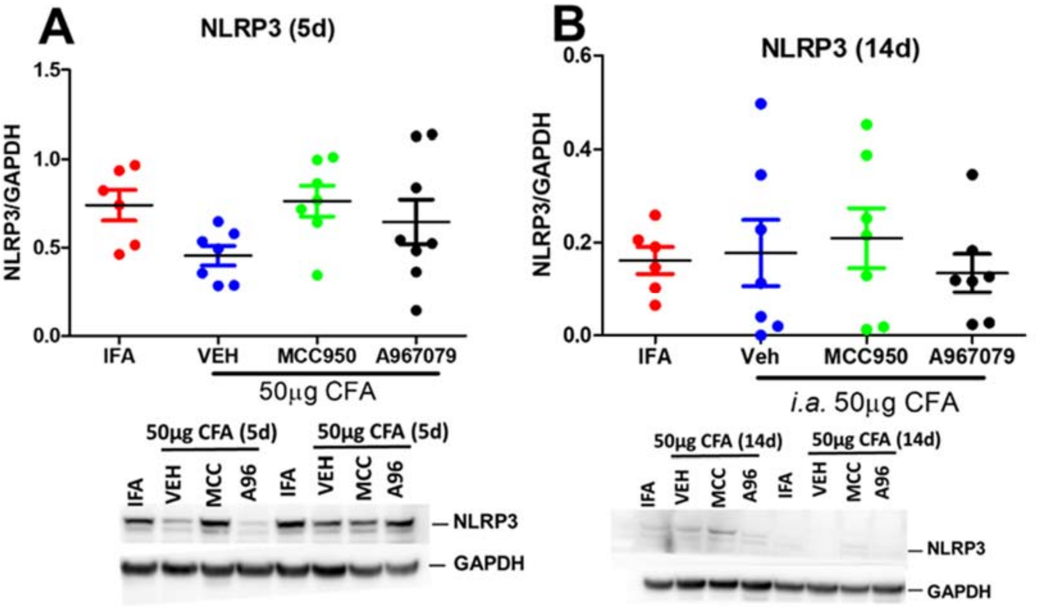

Supplementary Figure S1: Western blotting for NLRP3 expression in CFA-injected TMJs collected at days 5 or 14 in response to repeated treatments with a TRPA1 antagonist (A967079) or an NLRP3 antagonist (MCC950). A, B) NLRP3 in TMJs collected at days 5 or 14. Below are the representative blots Data represent mean±SEM, n= 7-8 animals per group. Data analysed by one-way ANOVA with Bonferroni's *post hoc* test.

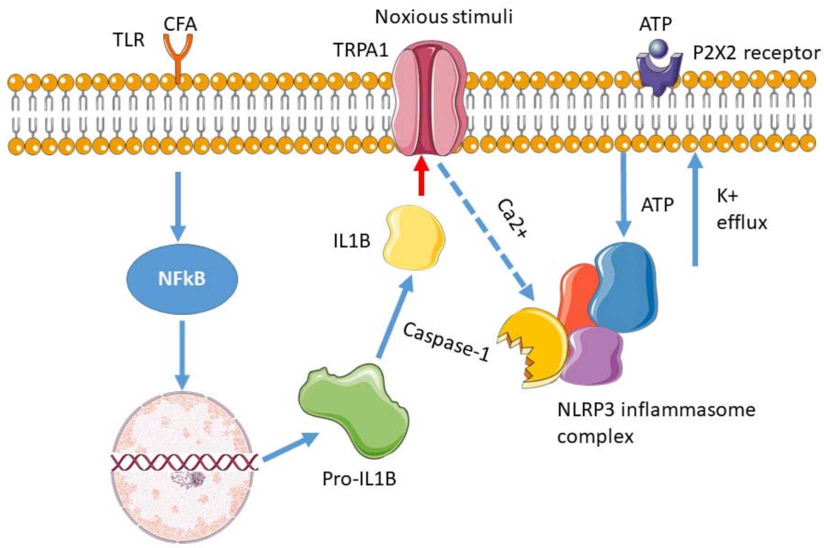

Supplementary Figure S2: Proposed cross talks of TRPA1 and NLRP3 inflammasome in CFA induced TMJ inflammation and pain. CFA activate TLR to induce NF-κB activation, expression of pro-IL1β and priming of NLRP3 inflammasome. Inflammasome is then activated by danger signals released from tissue injury such as ATP to release caspase-1 which in turn cleave pro-IL1β to produce active IL-1β which is released from the cells to sensitize TRPA1 in nociceptors and increase pain. Activation of TRPA1 result in release of intracellular calcium that may also activate NLRP3 inflammasome to create of vicious circle of inflammation and pain.
